# Structural Characterization of LsrK as a Quorum Sensing Target and a Comparison between X-ray and Homology Models

**DOI:** 10.1021/acs.jcim.0c01233

**Published:** 2021-03-08

**Authors:** Prasanthi Medarametla, Thales Kronenberger, Tuomo Laitinen, Antti Poso

**Affiliations:** † School of Pharmacy, Faculty of Health Sciences, University of Eastern Finland, P.O. Box 1627, FI-70211 Kuopio, Finland; ‡ Department of Oncology and Pneumonology, Internal Medicine, University Hospital Tübingen, Otfried-Müller-Straße 10, DE 72076 Tübingen, Germany

## Abstract

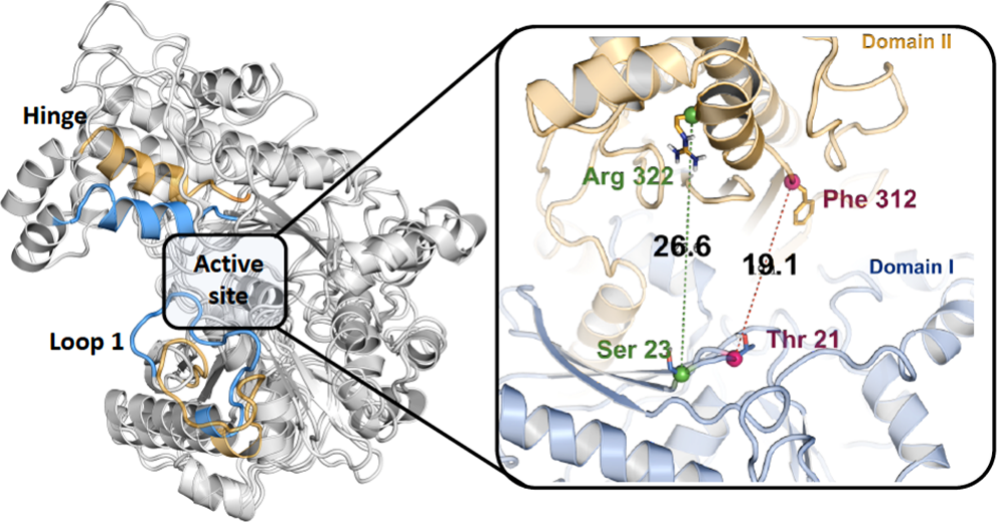

Quorum sensing is being investigated as an alternative therapeutic strategy in antibacterial drug discovery programs aimed at combatting bacterial resistance. LsrK is an autoinducer-2 kinase (belongs to the sugar kinase family), playing a key role in the phosphorylation of the autoinducer-2 (AI-2) signaling molecules involved in quorum sensing. Inhibiting LsrK could result in reduced pathogenicity by interfering with quorum sensing signaling. Previously, we have generated homology models to employ in structure-based virtual screening and successfully identified the first class of LsrK inhibitors. While conducting these studies, the crystal structure of LsrK was released, providing us with an opportunity to evaluate the reliability and quality of our models. A comparative structural analysis of the crystal structure and homology models revealed consistencies among them in the overall structural fold and binding site. Furthermore, the binding characteristics and conformational changes of LsrK have been investigated using molecular dynamics to inspect whether LsrK undergoes similar conformational changes as that of sugar kinases. These studies revealed the flexibility of the LsrK C-terminal domain (Domain II) attributing to the conformational changes in LsrK resulting in open and closed states during the phosphorylation. Further, simulations provided us with insights into the flexibility of a loop in Domain I that can influence the ligand accessibility to the LsrK binding site.

## Introduction

Quorum sensing (QS) is the process used by bacteria to communicate both between and within species. This communication controls population-based behaviors and functions such as virulence factor secretion, biofilm formation, motility, bioluminescence, sporulation, and the development of genetic competence.^1,2^ The QS process is mediated by the signaling molecules called autoinducers (AIs). These signaling molecules can be divided into three major groups: acylated homoserine lactones (AHL), autoinducer peptides (AIPs), and autoinducer-2 (AI-2). AHLs are *N*-acyl-l-homoserine lactones varying in their acyl chain length between 4 and 18 carbon atoms, while AIPs are oligopeptides. Generally, AIPs are utilized by gram-positive bacteria, whereas AHLs are used by gram-negative bacteria.^3^ In contrast, AI-2 molecules are used by both the gram-positive and gram-negative bacteria.^4,5^

These AI-2 molecules are synthesized by the LuxS family proteins by catalyzing the production of 4,5-dihydroxy-2,3-pentanedione (DPD) from *S*-ribosylhomocysteine.^6^ The produced AI-2 is then internalized from the extracellular environment into other bacterial cells by an adenosine triphosphate (ATP) binding cassette (ABC) transporter system called the Lsr transporter.^7^ Inside the cell, autoinducer-2 kinase, also known as LsrK kinase, phosphorylates the AI-2 molecules. The phosphorylated AI-2 undergoes isomerization by LsrF^8^ and LsrG,^9^ which further is responsible for the activation of the *lsr* operon and the inactivation of a repressor protein, LsrR.^7^ The *lsr* operon activation leads to the virulence factor secretion and biofilm formation causing the host pathogenicity. Thus, impairing the phosphorylation of AI-2 and inactivation of the *lsr* operon seems a promising strategy in the antibacterial drug discovery.^10^

The role of LsrK in AI-2 signaling was established in organisms such as *Escherichia coli*,^11^*Salmonella typhimurium,*^7,12^ and *Vibrio harveyi* through lsrK defective mutant studies. Further, the detailed AI-2 phosphorylation by LsrK kinase and enzyme kinetics were described by Zhu et al. using substrate specificity studies.^13^ This study also detailed the potentiality of developing DPD analogues as LsrK inhibitors. Although several DPD analogues were reported for the QS modulation, their mechanism and target of action were not described. There are only a few studies reporting LsrK inhibitors and DPD analogues that act specifically on LsrK.^14−17^ We recently have identified the first LsrK inhibitors using a homology model-based virtual screening method,^14^ whereas other inhibitors are from the high throughput screening (HTS) studies^15,16^ and substrate-based synthetic analogues.^17,18^

**Figure 1:**
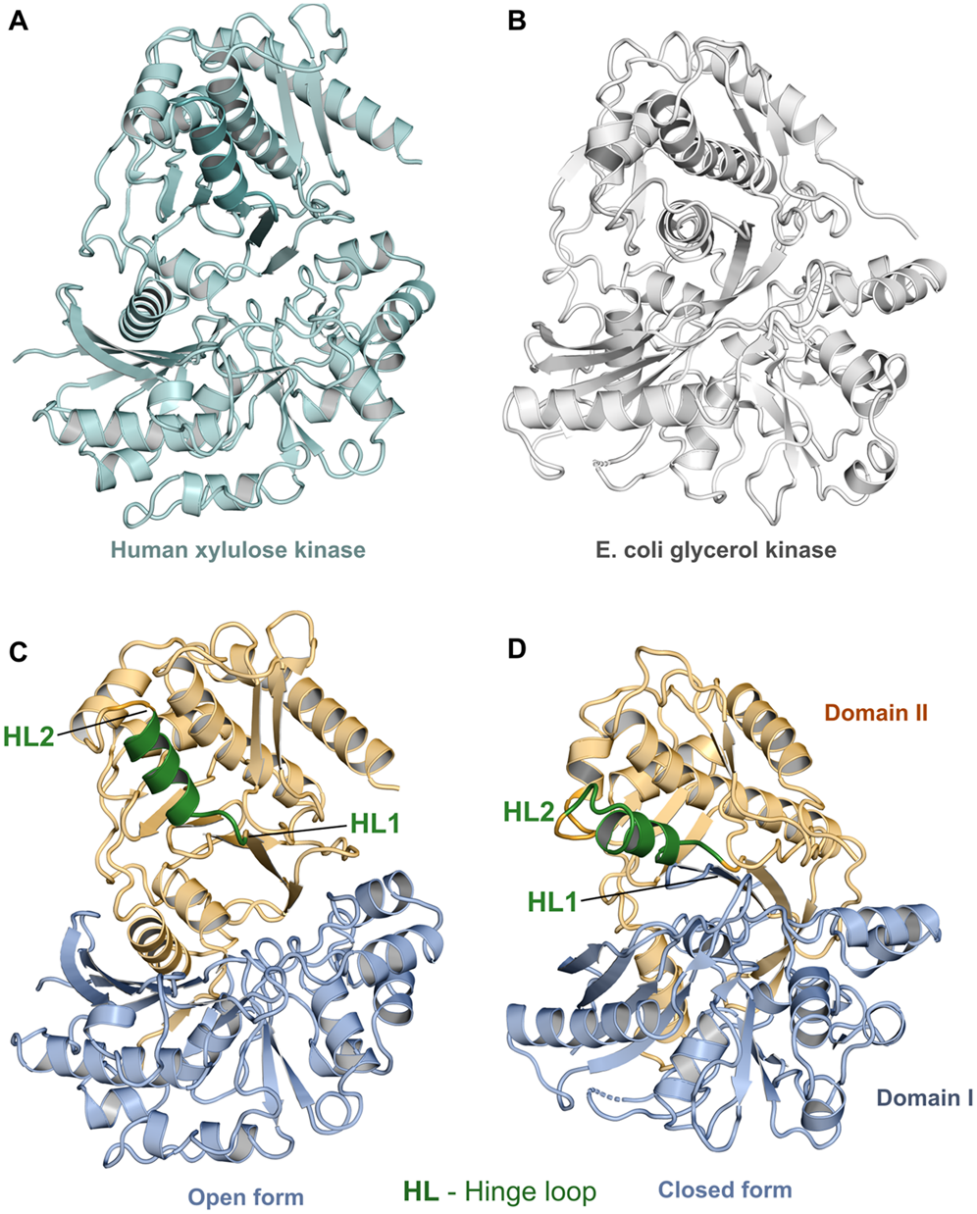
Crystal structures of carbohydrate kinase family proteins: human xylulose kinase (PDB ID: 4BC2) in the open form (A, C) and *E. coli* glycerol kinase (PDB ID: 1GLC) in the closed form (B, D). Interdomain movements and the hinge region are highlighted.

The structure of LsrK was not known until 2018 when the crystal structure of *E. coli* LsrK (open form) was released including a modulator protein called HPr (PDB ID: 5YA0, 5YA1, and 5YA2).^19^ Structurally, LsrK belongs to the FGGY carbohydrate kinases (sugar)^20^ consisting of two domains: Domain I (of N-terminal residues 13–260) and Domain II (of C-terminal residues 270–468), adopting the ribonuclease H-like fold.^21^ The sugar kinases are reported to show large interdomain movements during the catalysis, adopting an open or a closed form, which depends on the movement of (the hinge region) Domain II toward Domain I (Figure 1).

This movement has been shown clearly in the case of *E. coli* glycerol kinase,^22^*E. coli* xylulose kinase,^23^ and human xylulokinase^24^ (Figure 1). Based on this homologous structural data, we have generated two LsrK (*S. typhimurium*) homology models in the open and closed forms using xylulose kinase and glycerol kinase as templates, respectively.^14^

The main focus of the present study was to compare our earlier published homology models^14^ with the novel crystal structures.^19^ Furthermore, molecular dynamic (MD) simulations were employed to investigate whether LsrK adopts the conformational changes like other sugar kinases that are mentioned above (open and closed forms). Also, interdomain changes that have been reported to be existing in the sugar kinases were explored using MD in the case of LsrK. These structural details provided an understanding of the LsrK structure and the conformational changes that should be taken into account during the structure-based inhibitor design, targeting the LsrK kinase as a mechanism of interfering with the quorum sensing process.

## Methods

### Structure Preparation

#### Homology Models

The LsrK of *S. typhimurium* (stLsrK) is of 530 amino acids length (Uniprot ID: Q8ZKQ6). The stLsrK structure was modeled based on the homology with other FGGY carbohydrate kinase family proteins. Models were built in two conformations to mimic sugar kinases conformational changes of the open and closed forms using the templates xylulose kinase (PDB ID: 3HZ6) and glycerol kinase (PDB ID: 1GLC, chain G), respectively. The sequence alignment of LsrK and the templates was carried out in the Prime module of Schrödinger suite (Schrödinger release 2015-3: Prime, Schrödinger, LLC, New York, NY, 2015) and then manually edited with the help of multiple sequence alignment. Both models were generated using Prime including the cocrystallized ligands from the templates. Further, the prime refinement protocol was used to predict the side chains and minimized using OPLS_2005 force field.^25^ Validation of the models was done using tools such as Ramachandran Plot, ModVal, and ERRAT factor for their stereochemical quality and other parameters. In the Ramachandran plot, 98 and 94.8% residues were in the allowed regions of the open and closed models, respectively. The ERRAT quality factor of the open model was 82.7 and that of the closed model was 80.1. ModVal predicted that GA-341 was >0.7, which indicated that the models were reliable with a ≥95% probability of the correct fold. Based on these statistical parameters, homology models were found to be of optimum quality and these models were also used for the virtual screening studies. The basic methodology is described here and for more details, readers can refer to the previous publication.^14^

#### Crystal Structures of LsrK

*E. coli* LsrK (ecLsrK) (Uniprot ID: P77432, 530 amino acids long) was cocrystallized with a phosphoenolpyruvate (PEP)-dependent sugar phosphotransferase system (PTS) HPr protein.^19^ Three LsrK crystal structures with the HPr protein were deposited in the RCSB PDB: (i) the LsrK + HPr apo structure at 3.0 Å (PDB ID: 5YA0, hereafter referred to as CS-Open-Apo), (ii) LsrK+ HPr and ATP at 2.7 Å (PDB ID: 5YA1, hereafter referred to as CS-Open-ATP), and (iii) LsrK + HPr protein and adenosine diphosphate (ADP) (PDB ID: 5YA2, hereafter CS-Open-ADP) at 2.7 Å. It is noteworthy to mention that all of the three structures are in the open form.

The LsrK full-length sequences of *E. coli* (Uniprot ID: P77432) and *S. typhimurium* (Uniprot ID: Q8ZKQ6) were retrieved from UniProtKB and analyzed for the identity and similarity using the ClustalW alignment server and depicted using ENDScript 3.0 (Figure S1 in the Supporting Information). Next, to inspect the structural differences, crystal structures (5YA0, 5YA1, and 5YA2) were downloaded from the RCSB PDB. Protein structures (crystal structures and homology models) were prepared using the protein preparation wizard (PPW) module in Schrödinger (Schrödinger Release 2019-3: Protein Preparation Wizard; Epik, Schrödinger, LLC, New York, NY, 2019) to assign bond orders, fill missing side chains and loops, and generate ionization states. Here, missing residues (46–54) were added based on the sequence using the serial loop sampling method in the Prime module (Schrödinger Release 2019-3: Prime, Schrödinger, LLC, New York, NY, 2019). Furthermore, hydrogens were added, optimized, and then subjected to restrained minimization using the OPLS3e force field.^26^ To inspect the structural differences, the prepared crystal structures and homology models were aligned using the Schrödinger protein structure alignment tool based on the backbone Cα atoms and calculated the RMSD. Subsequently, the structures were visually inspected for the disparities near the ATP and substrate binding sites.

### Molecular Dynamics Simulations

Crystal structures (CS-Open-Apo, CS-Open-ATP, and CS-Open-ADP) and homology models (open and closed forms) were subjected to 500 ns simulations with the GPU-accelerated Desmond engine using the OPLS3e force field (Schrödinger Release 2019-3: Desmond, New York, NY, 2019). Simulated systems were composed of the solvated protein using the TIP3P solvent model within a 10 Å orthorhombic box under periodic boundary conditions and neutralized by placing ions of sodium (Na^+^). The systems were then equilibrated for 160 ps prior to the production runs using the default NPT ensemble relaxation protocol in Desmond. This includes several stages: (i) 100 ps of Brownian dynamics with an NVT ensemble at 10 K by posing restraints on solute heavy atoms; (ii) 12 ps simulations with the NVT ensemble using a Langevin thermostat (of 10 K) and restraints on solute heavy atoms; (iii) 12 ps simulations of the NPT ensemble using a Langevin thermostat (of 10 K), a Langevin barostat (of 1 atm), and restraints on solute heavy atoms; (iv) solvating the pocket; (v) 12 ps simulations with the NPT ensemble using a Langevin thermostat (of 300 K), a Langevin barostat (of 1 atm), and restraints on solute heavy atoms; and (vi) 24 ps simulations with the NPT ensemble using a Langevin thermostat (of 300 K), a Langevin barostat (of 1 atm), and no restraints. Production runs were carried out for 500 ns using an NPT ensemble at 310 K with the Nosé–Hoover chain Langevin thermostat method and a pressure of 1.01 bar using the Martyna–Tobias–Klein barostat method. Initial atom velocities were assigned by randomization. Coulombic interactions were explicitly calculated within a cutoff value of 9 Å. The RESPA-based integration method was used with a 2.0 fs timestep and structures were saved for every 100 ps for further analyses.

#### Trajectory Analysis

Primary trajectory analyses on all systems were carried out using the simulation interaction diagram (SID) tool in Desmond (Schrödinger Release 2019-3: Desmond, New York, NY, 2019). The root-mean-square deviation (RMSD) and root-mean-square fluctuations (RMSF) of the protein during the simulation were calculated based on the protein backbone Cα atoms. Protein–ligand interactions were also determined during the simulation using the SID. Further, to extract large conformational motions, essential dynamics analysis was performed using the traj_essential_dynamics.py script using backbone Cα atoms.^27^ Extreme protein motions generated from the traj_essential_dynamics.py script were visualized using the Modevectors script^28^ in PyMol 2.4.0 (PyMol Molecular Graphics System, Version 2.0 Schrödinger, LLC). Trajectories data is available in the Zenodo repository (under the code: 10.5281/zenodo.4511511).

#### Pocket Analysis

To investigate the changes in the binding site volume, initially, trajectory cluster analysis was performed using the Desmond trajectory clustering tool. Trajectory frames were clustered using the affinity propagation clustering method based on the RMSD of the protein backbone.^29^ Each trajectory was subjected to this clustering tool to generate 10 representative clusters, which were further utilized to evaluate the substrate binding site (pocket) volume using the SiteMap module in KNIME workflows.^30^ These structures were further clustered based on the volume overlap of the binding site.

#### Distance and Angle Calculations

A Schrödinger simulation event analysis tool was used to measure the residue distances and angles during the simulation. Residue distance measurements were carried out based on Cα atoms of the residues of Thr21, Ser23, and Thr456 of Domain I and Phe312, Arg322, and Phe325 of Domain II.

All of the graphics in this manuscript were generated using PyMol 2.4.0 (PyMol Molecular Graphics System, Version 2.4.0 Schrödinger, LLC).

## Results and Discussion

### Overall Protein Architecture

**Figure 2:**
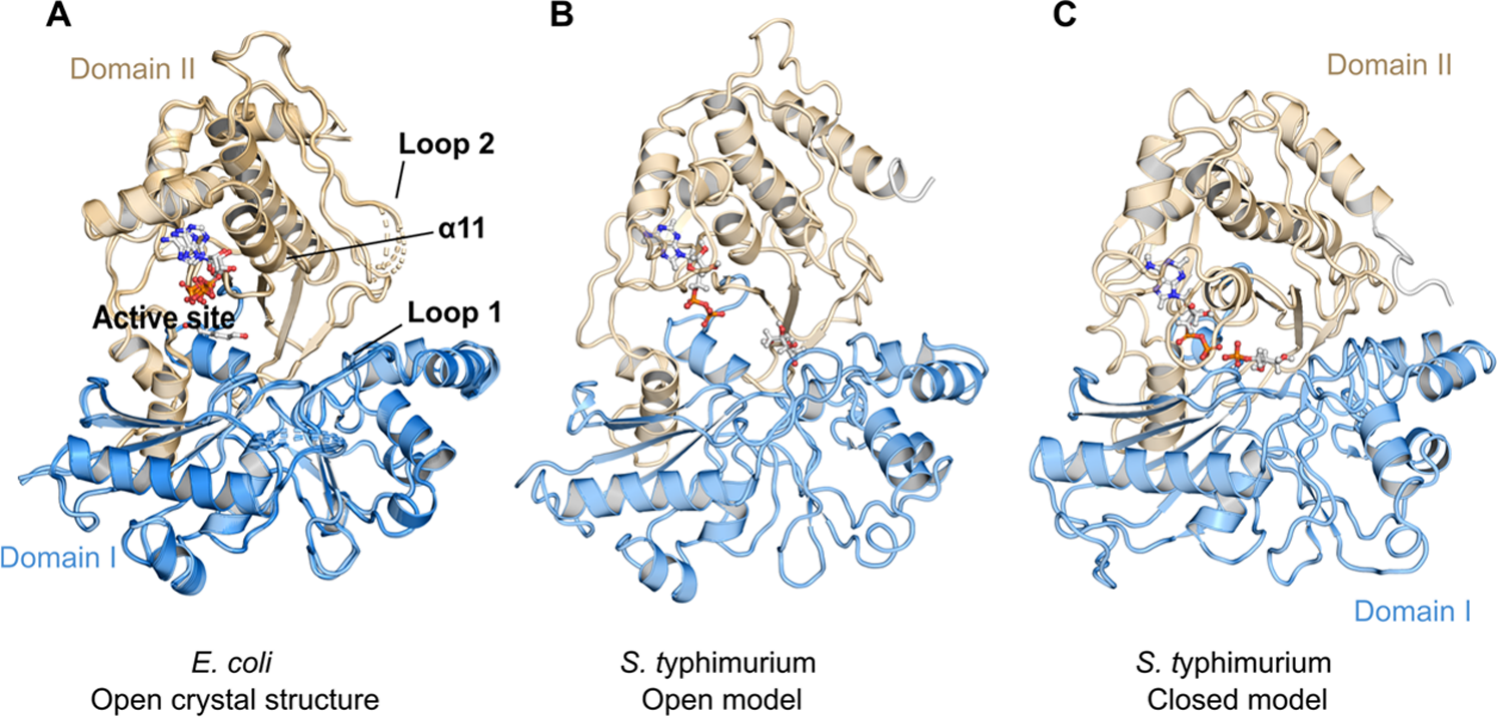
Structures of LsrK shown as cartoons: Domain I (blue) and Domain II (orange). (A) Alignment of the crystal structure of ecLsrK including ATP and ADP (PDB ID: 5YA0, 5YA1, and 5YA2); (B) stLsrK: an open model with xylulose and ADP; and (C) stLsrK: a closed model with glyceraldehyde-3-phosphate and ADP. Substrates ATP and ADP are displayed as sticks.

*E. coli* LsrK (ecLsrK) crystal structures (PDB ID: 5YA0, 5YA1, and 5YA2) and the generated homology models of stLsrK (*S. typhimurium* LsrK) are presented in Figure 2. The structure consists of two domains: Domain I (of residues 13–260) or N-terminal domain (NTD), and Domain II (of residues 270–468) or C-terminal domain. The active site is located in the cleft between Domain I and Domain II.^19^ LsrK structural homologs (sugar kinase family members) were found to exist in two conformations during the catalysis. Structural and mechanistic studies demonstrated that phosphorylation occurs in the closed form, i.e., the active state.^23^ In PDB, xylulose kinases (PDB ID: 2ITM and 2NLX (*E. coli*),^23^ 4BC2 (*H. sapiens*),^24^ and 3HZ6 (*Chromobacterium violaceum*)) exist in open conformation and glycerol kinase (PDB ID: 1GLC (*E. coli*)) in closed conformation.^31^ Conformational differences between xylulose kinase (PDB ID: 2ITM) and glycerol kinase (PDB ID: 1GLC)^23^ can be seen in Domain I in the vicinity of the substrate binding site (Figure 1C). ecLsrK crystal structures are in the open conformation and stLsrK homology models were generated to represent both the open and closed states. Further, ecLsrK and stLsrK structures were aligned to inspect the structural similarities and disparities.

### LsrK Structural Comparison

**Figure 3:**
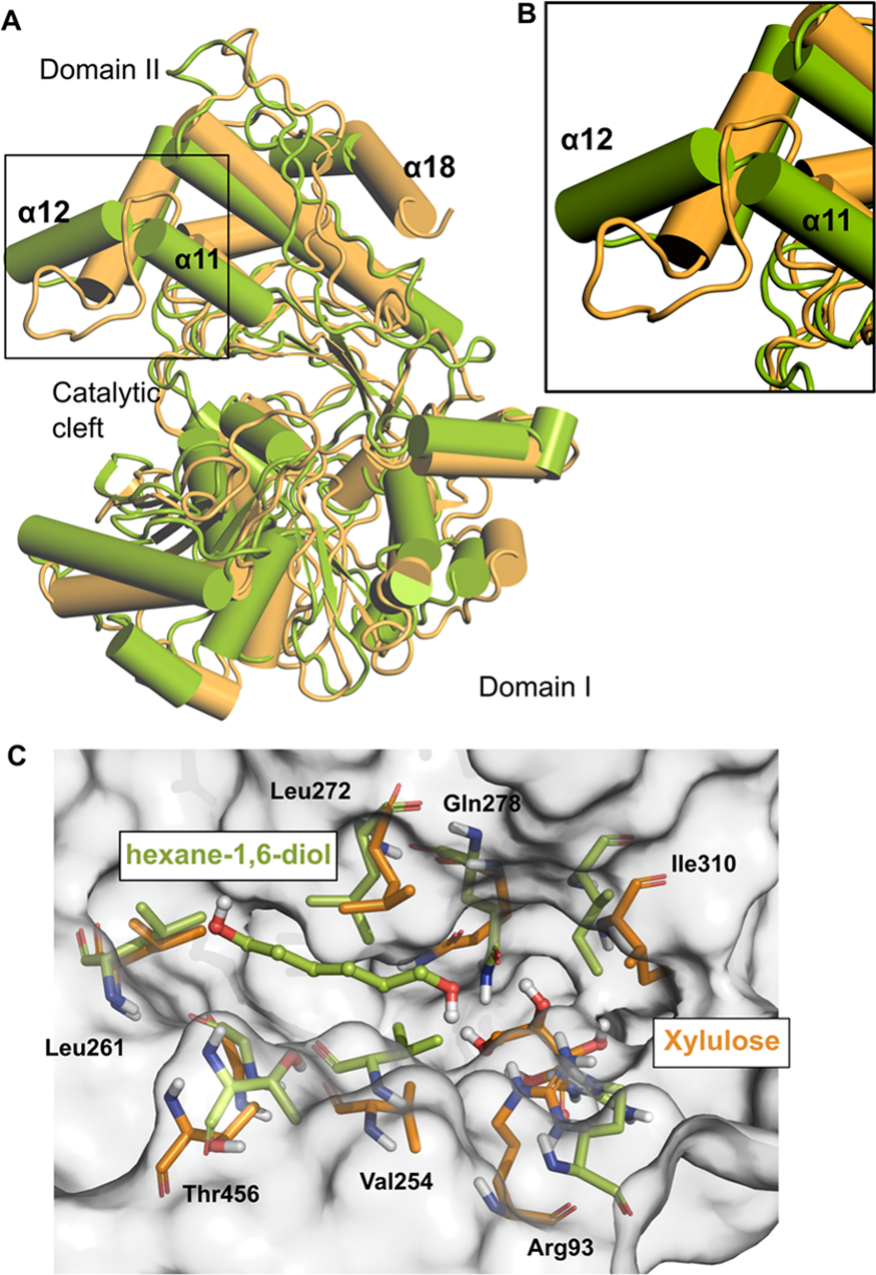
(A) Comparison of the homology model (HM-Open-ATP: orange color) and the X-ray crystal structure (CS-Open-ATP: green color). Secondary structural differences are shown: α12 that has been predicted as a loop (B) in the model (square box) and C-terminal helix α18. (C) Active site residues near the substrate binding site.

Sequence identities between *ec*LsrK and *st*LsrK were found to be 82.64%. The major sequence variations were found to be in Domain I of residues 76–85 and in Domain II of residues 419–424 and 496–503. The structural alignment of LsrK crystal structures and homology models displayed that the ecLsrK ATP-bound structure (PDB ID: 5YA1) and the stLsrK open model are aligned with overall backbone RMSD values of 2.89 and 0.97 Å for the binding site residues, respectively. Visual inspection revealed that secondary structural elements (structure helices, strands, and loops) are in good agreement with the ecLsrK crystal structure except for helix α12 of residues 326–337 (Figure 3A and see Figure S2 in the Supporting Information for numbering) and α18 (terminal residues of C-terminal domain or Domain II). Residues 326–337 (α12) were predicted to be a loop in the homology model, whereas in the crystal structure, these residues are part of a helix and located in the vicinity of the ATP binding site. However, none of the residues in this helix interact with ATP. The conserved substrate binding site residues were in similar conformation in the crystal structure and the homology model (Figure 3B).

### Conformational Changes and the Binding Site

The crystal structures of ecLsrK (PDB ID: 5YA0 (CS-Open-Apo), 5YA1 (CS-Open-ATP), and 5YA2 (CS-Open-ADP)) are in open conformation indicating the inactive state,^19^ and there are no major conformational differences observed among these structures. Homology models are in two conformations, i.e., open and closed forms, mimicking the inactive and active states.^26^ During the 500 ns MD simulations, hexane-1,6-diol (cryoprotectant comparable to the substrate) in CS-Open-ATP and xylulose (substrate) in Open-ATP were unstable in the binding site (Figures S3 and S4 in the Supporting information). This situation was in contrast to the case of Closed-ADP where the substrate (glyceraldehyde-3-phosphate) was stable in the binding site during the 500 ns timescale. This can be attributed to the interactions that occur between ADP and the substrate in the closed model during the simulation (Figure S4 in the Supporting information).

**Figure 4:**
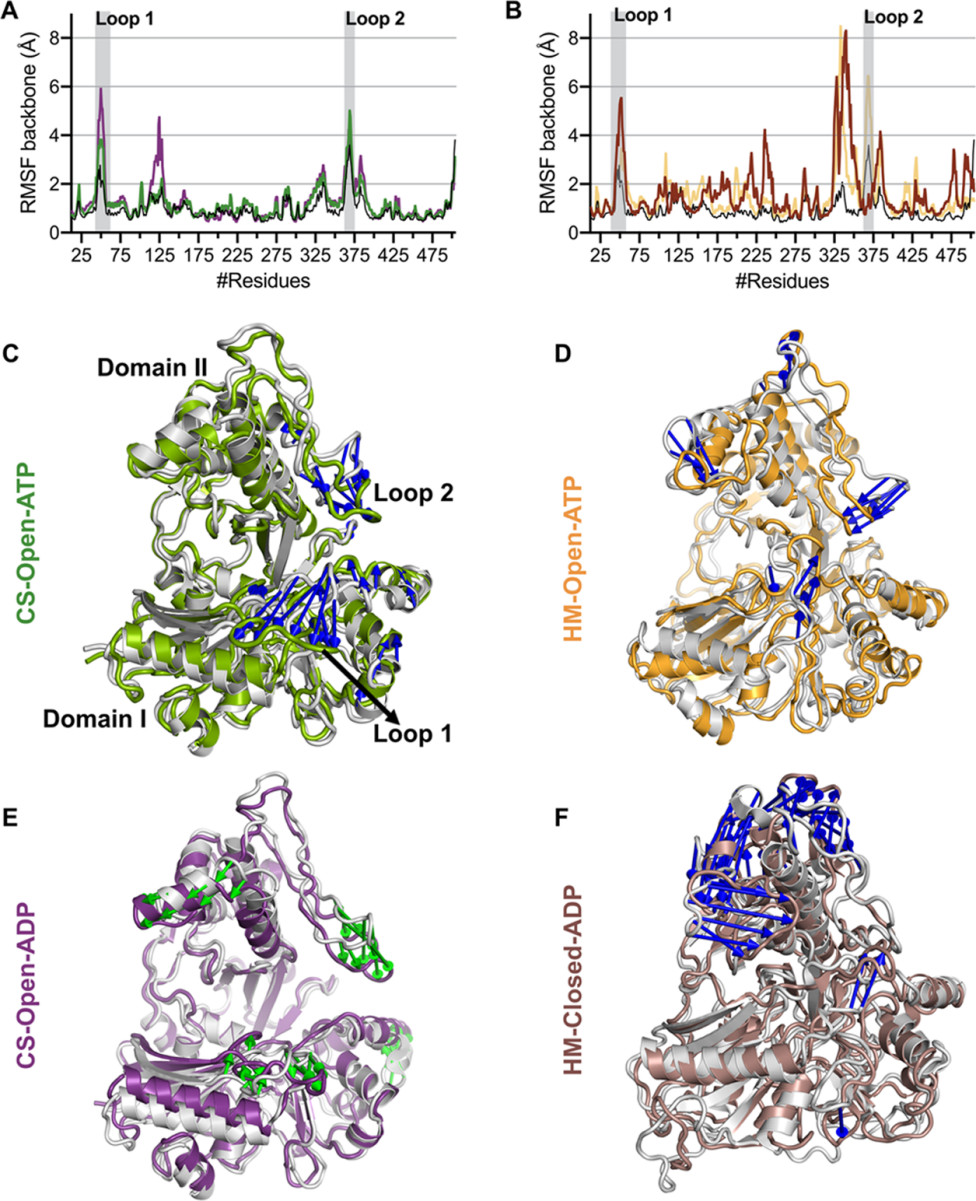
(A) RMSF of crystal structures during the 500 ns timescale. (B) RMSF of homology models during the 500 ns timescale. The extreme movements are represented by colored arrows, where the major fluctuating regions in all systems (C, D, E, and F) are contained within loop 1 (residues 45–62) and loop 2 (363–375).

Further, simulation data was exploited to retrieve the information on protein structural movements. Root-mean-square fluctuation (RMSF) values were used to identify flexible regions in the protein (Figure 4A,B). Generally, N- and C-terminal residues, as well as loop regions, show higher fluctuations compared with other secondary structural elements such as helices and strands. During the simulation, CS-Open-ATP displayed higher fluctuations (RMSF > 4 Å) in the loop regions of residues 45–62 (loop 1 highlighted with the gray color in Figure 4A,B) in Domain I (located between α1 and β3) and 360–378 (loop 2) in Domain II (located between α14 and α15). In addition to these loops, CS-Open-ADP has also shown noticeable movements near the turn residues 123–128 of Domain I [helix(α2)-turn-helix(α3)] and a shift in the helix α12 of Domain II (for protein numbering, refer to Figure S2 in the Supporting information). Loop 1 and loop 2 are in close proximity to the binding site region, where loop 1 might be involved in the phosphorylation process. Open-ATP also showed high RMSF (>5 Å) in the loop 2 region and minor fluctuations (RMSF < 3 Å) in loop 1 and loop 3 (constituted by hinge loop 2 near helix α11). In addition, the predicted loop (corresponding to helix α12 and hinge loop 2 in CS-Open-ATP) is also observed to be highly variable in CS-Open-ADP, Open-ATP, and Closed-ADP. To further investigate the specifics of the highly dynamic regions and the extreme movements, essential dynamics analysis was carried out on all systems. In all of the simulated systems, dominant movements were observed in loop 1 (of Domain I) and helix α12 (near the ATP binding site) and loop 2 of Domain II (Figure 4). In addition to the loop movements, CS-Open-ATP and CS-Open-ADP exhibited movements near α2 helix-turn-helix in Domain I. Closed-ADP revealed extreme movements in Domain II and small-scale movements in Domain I (of loop 1 and loop proceeding α9) (Figure 4F). In both the crystal structures and homology models, there were no structural movements observed near the substrate binding site. However, loop 1 (residues 45–62), located in the proximity of the catalytic cleft, has shown flexibility, affecting the size of the binding pocket. This region might be playing the role of a gatekeeper during the substrate binding and the catalytic reaction in LsrK. Unfortunately, the electron density of this loop region was not sufficient to model this part in the crystal structure and is being generated in our protein preparation steps. The domain movements observed in LsrK using mode analysis and RMSF are consistent with the reported conformational changes in the sugar kinase family^23,24^ with the exception of loop 1 flexibility. It is quite probable that this loop 1 dynamics can affect the ligand entry and ligand binding to LsrK.

### Understanding the Domain Movements Using Pocket Size Analysis

The simulation trajectory clustering was performed based on the backbone RMSD using the Schrodinger trajectory clustering tool. To investigate the pocket size and volume of the substrate binding site, the resulting 10 clusters (centroids) were used. These clusters were subjected to pocket parameter prediction using SiteMap in Schrodinger KNIME workflows. SiteMap predicts possible druggable pockets and associated parameters such as size, volume, SiteScore, Dscore, hydrophobicity, and hydrophilicity. Here, a single binding site, i.e., the substrate binding site, was analyzed to predict the pocket parameters throughout the trajectory. Based on the predicted pocket volume, clusters were generated for all structures (CS-Open-Apo, CS-Open-ATP, CS-Open-ADP, HM-Open-ATP, and HM-Closed-ATP). All pocket parameters are tabulated in the Supporting Information (Tables S1–S5).

**Figure 5:**
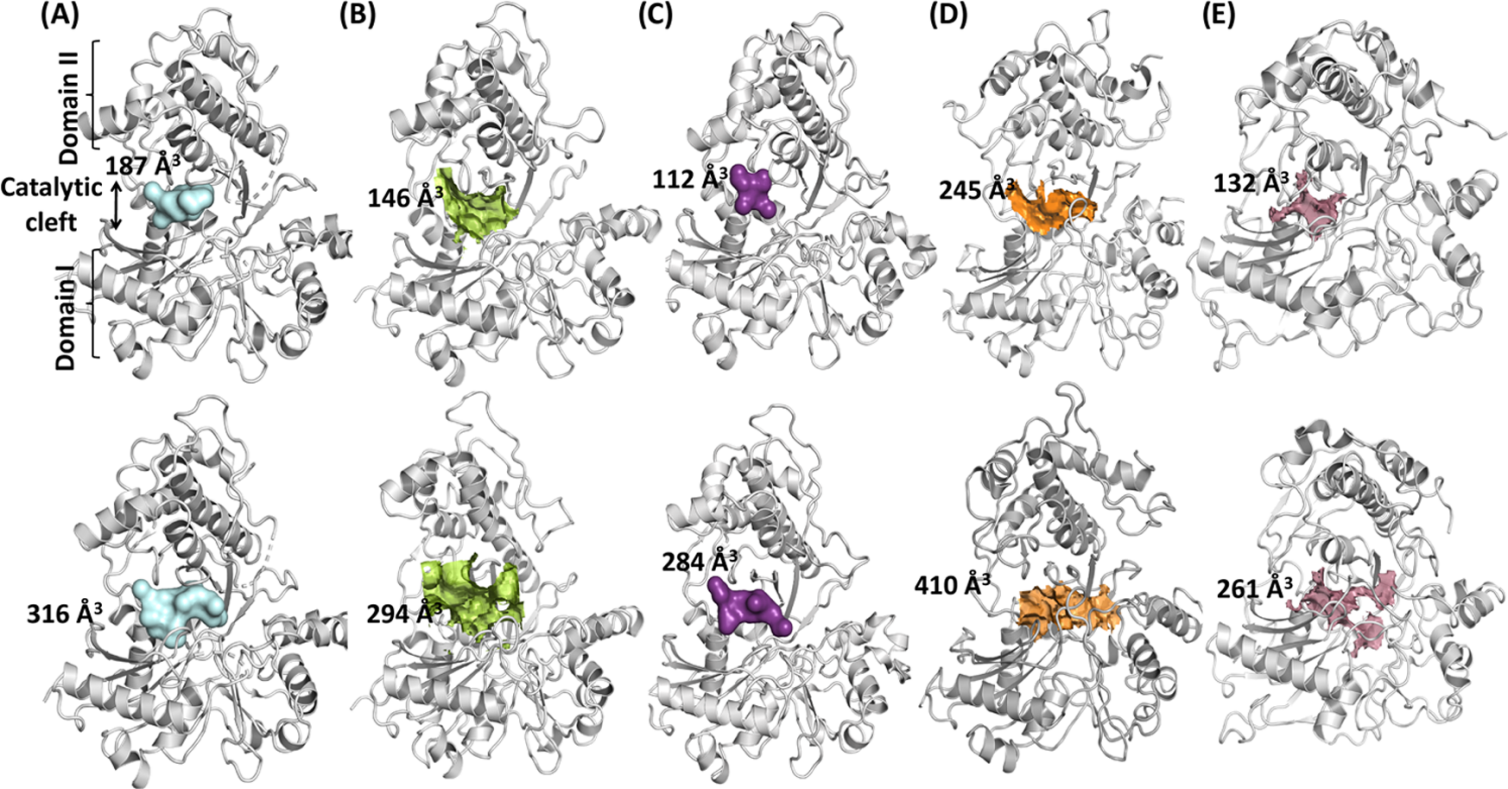
SiteMap predicted the substrate binding site volume (i.e., colored surface). The top row represents the lowest site volume and the bottom row indicates the highest site volume. (A) CS-Open-Apo, (B) CS-Open-ATP, (C) CS-Open-ADP, (D) HM-Open-ATP, and (E) HM-Closed-ADP.

**Figure 6:**
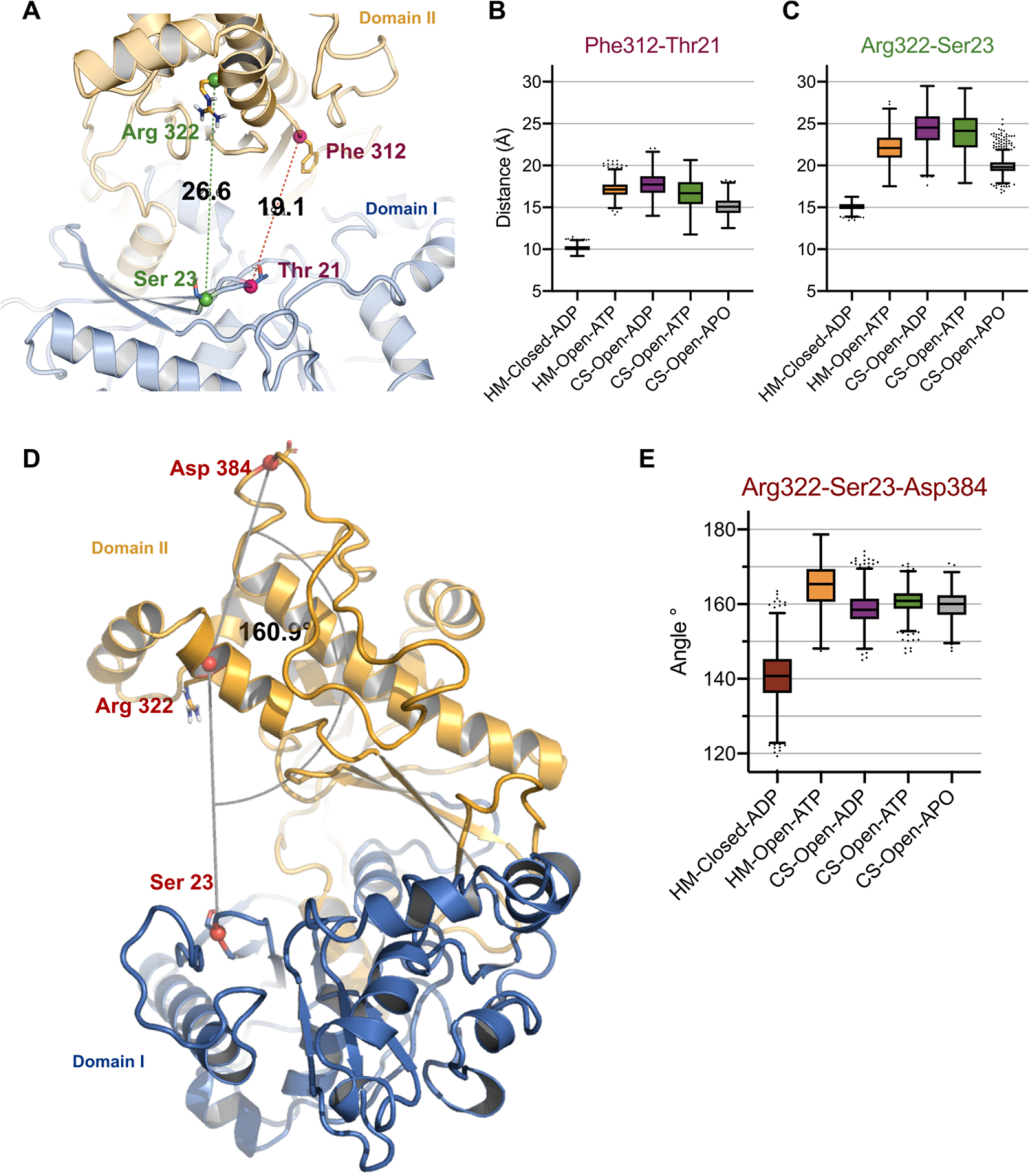
(A) Illustration of the interdomain residue distances in the crystal structure (CS-Open-ATP). Residue distance measurements calculated throughout the 500 ns trajectory simulation of all systems are presented (B, C). (D) Angle measurement of the interdomain residues in the reference structure (CS-Open-ATP). (E) Angle measurements of the interdomain residues during the 500 ns timescale. Coordinates used for the angle and distance measurements are depicted as spheres. The central box line represents the average, and the points represent Tukey determined outliers.

SiteMap pocket predictions revealed that the volume of the pocket in all systems varies throughout the simulation. For instance, the pocket volume changes from 190.7 to 294.63 Å^3^ in the CS-Open-ATP simulation (Figure 5B). The majority of pocket changes can be attributed to the movements of loop 1 and Domain II (movement toward Domain I). The pocket volume changed from 112.16 to 316.9 Å^3^ among the crystal structures (CS-Open-Apo, CS-Open-ATP, and CS-Open-ADP) and 132.1–410.2 Å^3^ among the homology models (HM-Open-ATP and HM-Closed-ADP). Further, to quantify the interdomain movements, distances were measured for residues surrounding the substrate binding site and the ATP binding site (Figure 6A). The original distance observed in the crystal structure between the substrate binding site residues Thr21 (of Domain I) and Phe312 (of Domain II) is 19 Å. This distance has been decreased to 16 Å through the trajectory of CS-Open-ATP and to 14 Å in CS-Open-ADP (Figure 6B). The distance between the ATP binding site residue Arg322 (representing the start of hinge loop 2 in Domain II) and Ser23 of the catalytic cleft (β loop of Domain I) was 26.6 Å (in the crystal structure). The decrease of this distance to 23 Å (Figure 6C) demonstrates the conformational change from the open state to the closed state. A similar trend was observed in CS-Open-ADP and also in HM-Open-ATP (Figure 6B,C). However, there are no major differences in the distances of HM-Closed-ADP. This might be resulting due to the tight packing of the atoms in the closed model and the presence of ligands (ADP and glyceraldehyde-3-phosphate) that make interactions with the surrounding residues during the simulation (Figure S4 in the Supporting Information). Further, we evaluated the domain movements in regard to the angle measurements between Domain I (Thr21) and Domain II (Arg322 and Asp384). The reference value in the CS-Open-Apo structure was 160.1° (Figure 6D). The relative position of domains has changed from 140° (HM-Closed-ADP) to 170° (HM-Open-ATP). The structural changes observed in LsrK during the dynamic simulations are consistent with the conformational flexibility observed in other sugar kinases (xylulose kinase and glycerol kinase).^23,32^ Earlier studies described only the rigid body motion of Domain II toward Domain I. In addition to these, our mode analysis and pocket volume predictions indicated the movement of loop 1 (of Domain I) that might influence the inhibitor binding to LsrK.

## Conclusions

LsrK is the key kinase involved in the quorum sensing process that regulates the virulence and pathogenicity in bacterial infections. Considering the role of LsrK as an antivirulence target, we have identified the first class of LsrK inhibitors employing the virtual screening study by generating homology models. The current study indicates the quality of our homology models because of their structural consistency with the recently released crystal structures of LsrK. Further, molecular dynamics simulations on these structures provided details of domain movements and structural flexibility that can help in structure-based drug design efforts to target the LsrK binding site.

Our results demonstrated that loop 1 (residues 45–62) conformational changes could influence the ligand entry and binding in the active site. Further, binding site volume changes provided the information that the LsrK binding site can accommodate large size ligands to small size ligands, depending on the protein conformation whether it is in the open form or the closed form. Although open and closed states and Domain II movements were established in the sugar kinases, loop 1 changes were not discussed. This loop flexibility was not explored in our prior virtual screening study. This new information can be a gain in the future virtual screening campaigns that can be taken into account for the LsrK inhibitor design. However, experimental studies are further needed to confirm and characterize the phosphorylation events occurring in the LsrK active site, as this information would further help in the LsrK-targeted drug design.

## Supporting Information Available

Sequence alignment of *E. coli* LsrK structure and *S. typhimurium* LsrK kinase (Figure S1); numbering for the reference of (Figure S2); protein (Cα atoms) RMSD and ligand RMSD of the crystal structure (Figure S3); RMSD of protein and ligand during the 500 ns simulation of homology (Figure S4); RMSD of the LsrK structure through the 500 ns simulation trajectory in comparision with the Apo structure (Figure S5); Sitemap predicted substrate binding site parameters for the trajectory cluster centroids of crystal structures and homology models (Table S1–S5) (PDF)

## References

[ref1] Suga H.; Smith K. M. Molecular Mechanisms of Bacterial Quorum Sensing as a New Drug Target. Curr. Opin. Chem. Biol. 2003, 7, 586–591. 10.1016/j.cbpa.2003.08.001. 14580562

[ref2] Chen G.; Swem L. R.; Swem D. L.; Stauff D. L.; O’Loughlin C. T.; Jeffrey P. D.; Bassler B. L.; Hughson F. M. A Strategy for Antagonizing Quorum Sensing. Mol. Cell 2011, 42, 199–209. 10.1016/j.molcel.2011.04.003. 21504831 PMC3092643

[ref3] Fuqua W. C.; Winans S. C.; Greenberg E. P. Quorum Sensing in Bacteria: The LuxR-LuxI Family of Cell Density- Responsive Transcriptional Regulators. J. Bacteriol. 1994, 176, 269–275. 10.1128/jb.176.2.269-275.1994. 8288518 PMC205046

[ref4] Xavier K. B.; Bassler B. L. Interference with AI-2-Mediated Bacterial Cell–Cell Communication. Nature 2005, 437, 750 10.1038/nature03960. 16193054 PMC1388276

[ref5] Surette M. G.; Miller M. B.; Bassler B. L. Quorum Sensing in *Escherichia coli*, Salmonella Typhimurium, and Vibrio Harveyi: A New Family of Genes Responsible for Autoinducer Production. Proc. Natl. Acad. Sci. U.S.A. 1999, 96, 1639–1644. 10.1073/pnas.96.4.1639. 9990077 PMC15544

[ref6] Schauder S.; Shokat K.; Surette M. G.; Bassler B. L. The LuxS Family of Bacterial Autoinducers: Biosynthesis of a Novel Quorum-Sensing Signal Molecule. Mol. Microbiol. 2001, 41, 463–476. 10.1046/j.1365-2958.2001.02532.x. 11489131

[ref7] Taga M. E.; Miller S. T.; Bassler B. L. Lsr-Mediated Transport and Processing of AI-2 in Salmonella Typhimurium. Mol. Microbiol. 2003, 50, 1411–1427. 10.1046/j.1365-2958.2003.03781.x. 14622426

[ref8] Marques J. C.; Oh I. K.; Ly D. C.; Lamosa P.; Ventura M. R.; Miller S. T.; Xavier K. B. LsrF, a Coenzyme A-Dependent Thiolase, Catalyzes the Terminal Step in Processing the Quorum Sensing Signal Autoinducer-2. Proc. Natl. Acad. Sci. U.S.A. 2014, 111, 14235–14240. 10.1073/pnas.1408691111. 25225400 PMC4191781

[ref9] Marques J. C.; Lamosa P.; Russell C.; Ventura R.; Maycock C.; Semmelhack M. F.; Miller S. T.; Xavier K. B. Processing the Interspecies Quorum-Sensing Signal Autoinducer-2 (AI-2): Characterization of Phospho-(S)-4,5-Dihydroxy-2,3-Pentanedione Isomerization by LsrG Protein. J. Biol. Chem. 2011, 286, 18331–18343. 10.1074/jbc.M111.230227. 21454635 PMC3093905

[ref10] Mühlen S.; Dersch P. Anti-Virulence Strategies to Target Bacterial Infections. Curr. Top. Microbiol. Immunol. 2016, 398, 147–183. 10.1007/82_2015_490. 26942418

[ref11] Xavier K. B.; Bassler B. L. Regulation of Uptake and Processing of the Quorum-Sensing Autoinducer AI-2 in *Escherichia coli*. J. Bacteriol. 2005, 187, 238–248. 10.1128/JB.187.1.238-248.2005. 15601708 PMC538819

[ref12] Roy V.; Fernandes R.; Tsao C. Y.; Bentley W. E. Cross Species Quorum Quenching Using a Native AI-2 Processing Enzyme. ACS Chem. Biol. 2010, 5, 223–232. 10.1021/cb9002738. 20025244

[ref13] Zhu J.; Hixon M. S.; Globisch D.; Kaufmann G. F.; Janda K. D. Mechanistic Insights into the LsrK Kinase Required for Autoinducer-2 Quorum Sensing Activation. J. Am. Chem. Soc. 2013, 135, 7827–7830. 10.1021/ja4024989. 23672516 PMC3736694

[ref14] Medarametla P.; Gatta V.; Kajander T.; Laitinen T.; Tammela P.; Poso A. Structure-Based Virtual Screening of LsrK Kinase Inhibitors to Target Quorum Sensing. ChemMedChem 2018, 13, 2400–2407. 10.1002/cmdc.201800548. 30178912

[ref15] Gatta V.; Ilina P.; Porter A.; McElroy S.; Tammela P. Targeting Quorum Sensing: High-Throughput Screening to Identify Novel Lsrk Inhibitors. Int. J. Mol. Sci. 2019, 20, 3112 10.3390/ijms20123112. PMC6627609 31242708

[ref16] Gatta V.; Tomašič T.; Ilaš J.; Zidar N.; Peterlin Mašič L.; Barančoková M.; Frlan R.; Anderluh M.; Kikelj D.; Tammela P. A New Cell-Based AI-2-Mediated Quorum Sensing Interference Assay in Screening of LsrK-Targeted Inhibitors. ChemBioChem 2020, 21, 1918–1922. 10.1002/cbic.201900773. 32026533

[ref17] Stotani S.; Gatta V.; Medarametla P.; Padmanaban M.; Karawajczyk A.; Giordanetto F.; Tammela P.; Laitinen T.; Poso A.; Tzalis D.; Collina S. DPD-Inspired Discovery of Novel LsrK Kinase Inhibitors: An Opportunity To Fight Antimicrobial Resistance. J. Med. Chem. 2019, 62, 2720–2737. 10.1021/acs.jmedchem.9b00025. 30786203

[ref18] Linciano P.; Cavalloro V.; Martino E.; Kirchmair J.; Listro R.; Rossi D.; Collina S. Tackling Antimicrobial Resistance with Small Molecules Targeting LsrK: Challenges and Opportunities. J. Med. Chem. 2020, 63, 15243–15257. 10.1021/acs.jmedchem.0c01282. 33152241 PMC8016206

[ref19] Ha J. H.; Hauk P.; Cho K.; Eo Y.; Ma X.; Stephens K.; Cha S.; Jeong M.; Suh J.-Y.; Sintim H. O.; Bentley W. E.; Ryu K.-S. Evidence of Link between Quorum Sensing and Sugar Metabolism in *Escherichia coli* Revealed via Cocrystal Structures of LsrK and HPr. Sci. Adv. 2018, 4, eaar7063 10.1126/sciadv.aar7063. 29868643 PMC5983913

[ref20] Zhang Y.; Zagnitko O.; Rodionova I.; Osterman A.; Godzik A. The FGGY Carbohydrate Kinase Family: Insights into the Evolution of Functional Specificities. PLoS Comput. Biol. 2011, 7, e1002318 10.1371/journal.pcbi.1002318. 22215998 PMC3245297

[ref21] Cheek S.; Zhang H.; Grishin N. V. Sequence and Structure Classification of Kinases. J. Mol. Biol. 2002, 320, 855–881. 10.1016/S0022-2836(02)00538-7. 12095261

[ref22] Feese M. D.; Faber H. R.; Bystrom C. E.; Pettigrew D. W.; Remington S. J. Glycerol Kinase from *Escherichia coli* and an Ala65→Thr Mutant: The Crystal Structures Reveal Conformational Changes with Implications for Allosteric Regulation. Structure 1998, 6, 1407–1418. 10.1016/S0969-2126(98)00140-3. 9817843

[ref23] Di Luccio E.; Petschacher B.; Voegtli J.; Chou H.-T.; Stahlberg H.; Nidetzky B.; Wilson D. K. Structural and Kinetic Studies of Induced Fit in Xylulose Kinase from *Escherichia coli*. J. Mol. Biol. 2007, 365, 783–798. 10.1016/j.jmb.2006.10.068. 17123542 PMC1995121

[ref24] Bunker R. D.; Bulloch E. M. M.; Dickson J. M. J.; Loomes K. M.; Baker E. N. Structure and Function of Human Xylulokinase, an Enzyme with Important Roles in Carbohydrate Metabolism. J. Biol. Chem. 2013, 288, 1643–1652. 10.1074/jbc.M112.427997. 23179721 PMC3548474

[ref25] Banks J. L.; Beard H. S.; Cao Y.; Cho A. E.; Damm W.; Farid R.; Felts A. K.; Halgren T. A.; Mainz D. T.; Maple J. R.; Murphy R.; Philipp D. M.; Repasky M. P.; Zhang L. Y.; Berne B. J.; Friesner R. A.; Gallicchio E.; Levy R. M. Integrated Modeling Program, Applied Chemical Theory (IMPACT). J. Comput. Chem. 2005, 26, 1752–1780. 10.1002/jcc.20292. 16211539 PMC2742605

[ref26] Roos K.; Wu C.; Damm W.; Reboul M.; Stevenson J. M.; Lu C.; Dahlgren M. K.; Mondal S.; Chen W.; Wang L.; Abel R.; Friesner R. A.; Harder E. D. OPLS3e: Extending Force Field Coverage for Drug-Like Small Molecules. J. Chem. Theory Comput. 2019, 15, 1863–1874. 10.1021/acs.jctc.8b01026. 30768902

[ref27] Grant B. J.; Rodrigues A. P. C.; ElSawy K. M.; McCammon J. A.; Caves L. S. D. Bio3d: An R Package for the Comparative Analysis of Protein Structures. Bioinformatics 2006, 22, 2695–2696. 10.1093/bioinformatics/btl461. 16940322

[ref28] Sean M. PyMOL Script: modevectors.py, 2020.

[ref29] Frey B. J.; Dueck D. Clustering by Passing Messages between Data Points. Science 2007, 315, 972–976. 10.1126/science.1136800. 17218491

[ref30] Halgren T. A. Identifying and Characterizing Binding Sites and Assessing Druggability. J. Chem. Inf. Model. 2009, 49, 377–389. 10.1021/ci800324m. 19434839

[ref31] Feese M.; Pettigrew D. W.; Meadow N. D.; Roseman S.; Remington S. J. Cation-Promoted Association of a Regulatory and Target Protein Is Controlled by Protein Phosphorylation. Proc. Natl. Acad. Sci. U.S.A. 1994, 91, 3544–3548. 10.1073/pnas.91.9.3544. 8170944 PMC43616

[ref32] Hurley J. H.; Faber H. R.; Worthylake D.; Meadow N. D.; Roseman S.; Pettigrew D. W.; Remington S. J. Structure of the Regulatory Complex of *Escherichia coli* IIIGlc with Glycerol Kinase. Science 1993, 259, 673–677. 10.1126/science.8430315. 8430315

