# Supplementary material for: Structural Characterization of LsrK as a Quorum Sensing Target and a Comparison between X-ray and Homology Models
Source: J Chem Inf Model. 2021 Mar 8;61(3):1346–53. doi: 10.1021/acs.jcim.0c01233 (PMC8028047; doi:10.1021/acs.jcim.0c01233)
Supplement: Supplementary file 1 — ci0c01233_si_001.pdf [file ci0c01233_si_001.pdf]

Structural characterization of LsrK as a quorum sensing target and a comparison between X-ray and homology models

Prasanthi Medarametla[1], Thales Kronenberger[1,2], Tuomo Laitinen[1], Antti Poso[1,2*]

1. School of Pharmacy, Faculty of Health Sciences, University of Eastern Finland, P.O. Box 1627, FI-70211 Kuopio, Finland email: antti.poso@uef.fi
2. Department of Oncology and Pneumonology, Internal Medicine, University Hospital Tübingen, Otfried-Müller-Straße 10, DE 72076 Tübingen, Germany

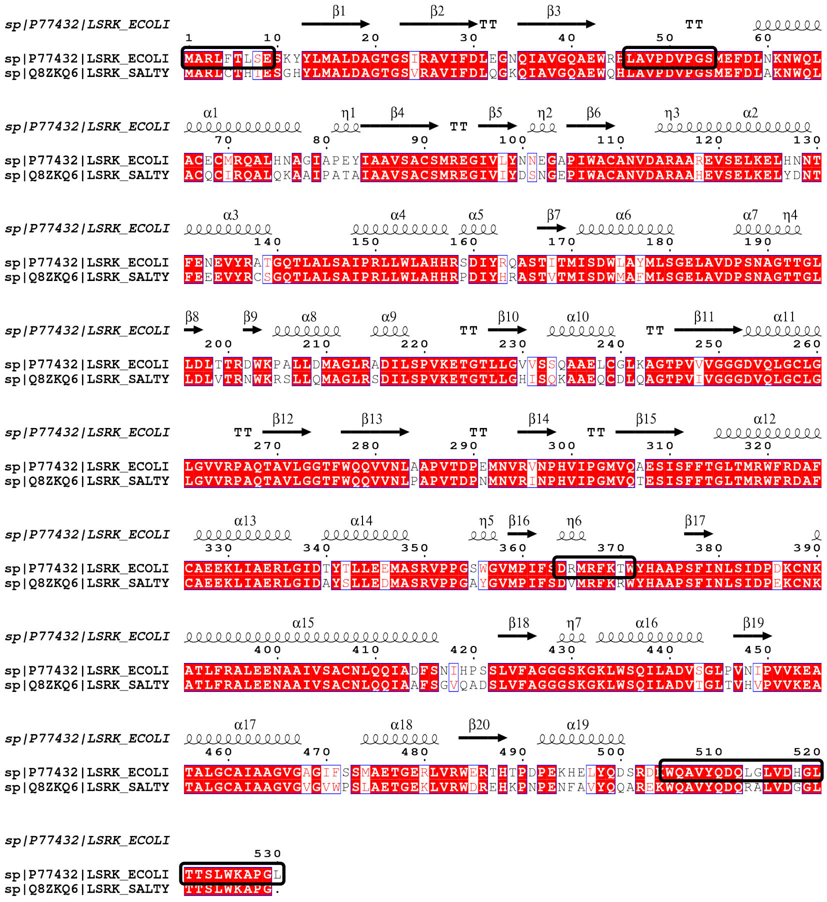

Figure S1. Sequence alignment of E. coli LsrK kinase and S. typhimurium LsrK kinase. Red shading shows the identical residues. Secondary structural elements of the crystal structure were shown above the alignment. α-Helices were shown as squiggles and β-strands as arrows, strict β-turns as TT and strict α-turns as TTT. Regions that were not identified in the X-ray structure are highlighted with black boxes.

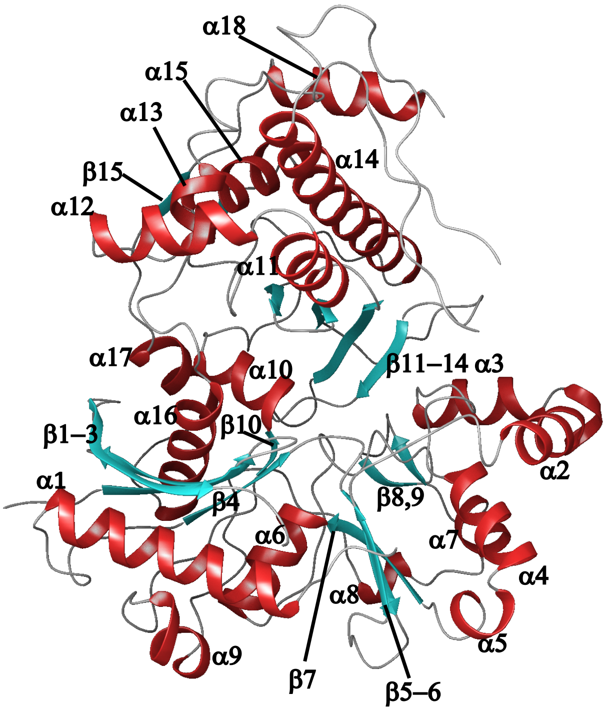

Figure S2. Numbering for the reference of structure of LsrK (PDBID: 5YA1)

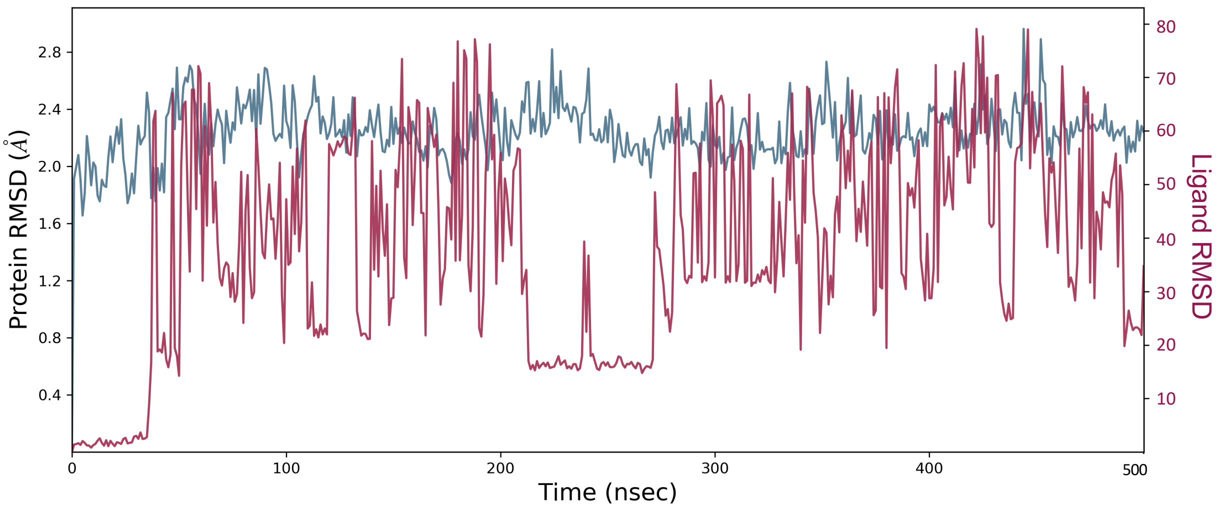

Figure S3. Protein (Cα-atoms) RMSD and ligand RMSD of crystal structure (5YA1:CS-Open-ATP) during the dynamic simulations. RMSD is shown during the simulation timescale. Blue lines in the graph indicate protein RMSD and brown lines indicate ligand RMSD with respect to the protein.

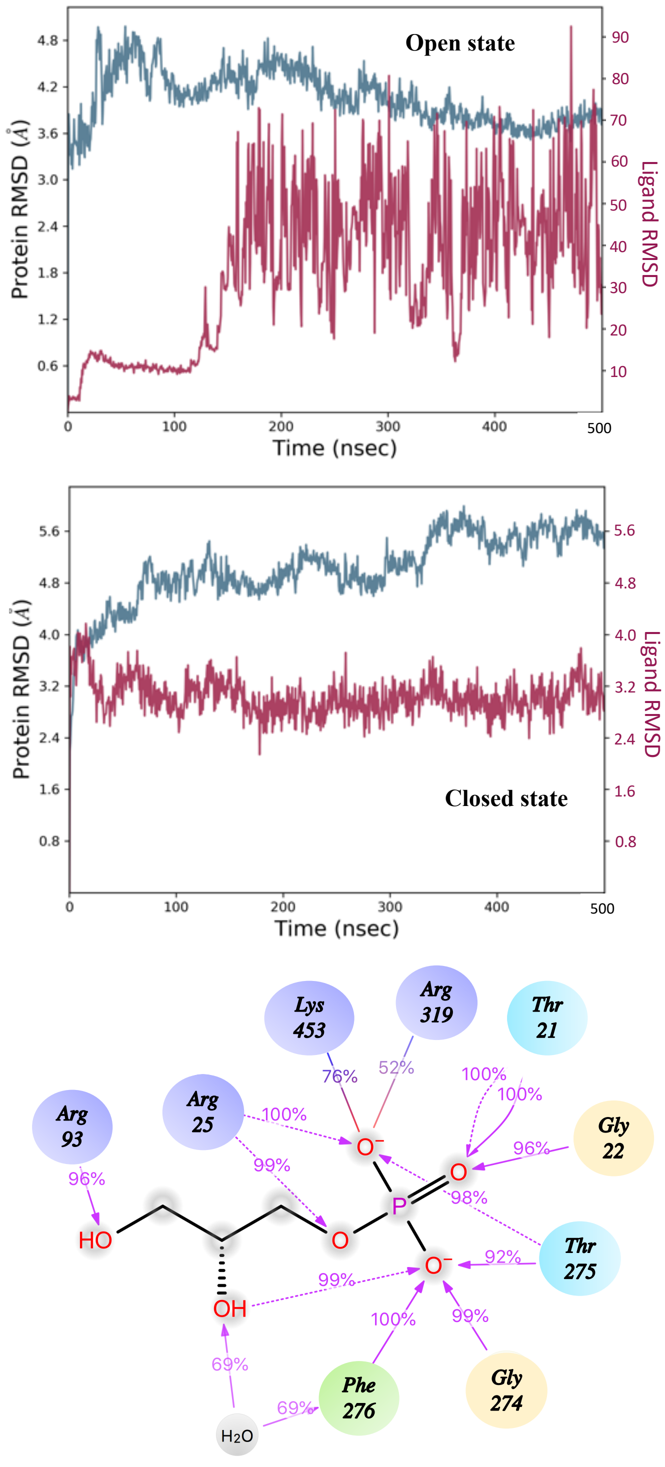

Figure S4. RMSD of protein and ligand during the 500ns simulations of homology models. Protein-ligand interaction diagram of the Closed-ADP during the 500ns simulation time scale. Interactions and time of residence (%) are depicted.

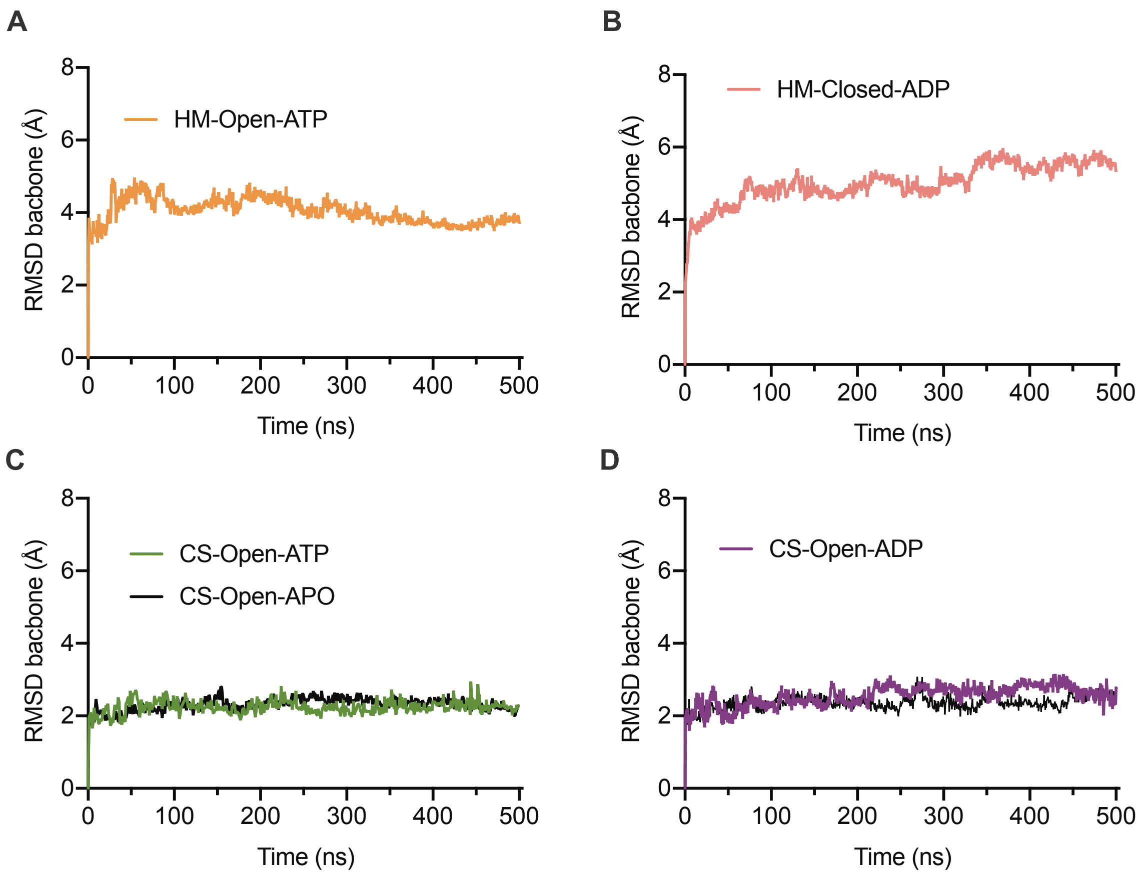

Figure S5. RMSD of LsrK structure through 500 ns simulation trajectory.

Table S1. Sitemap predicted pocket parameters for the trajectory cluster centroids of ATP and substrate bound crystal structure (5YA0 or CS-Open-Apo).

Cluster Pocket Number	Size	Volume
Pocket 1	60	300.12
Pocket 2	67	187.96
Pocket 3	88	252.44
Pocket 4	69	294.98
Pocket 5	89	316.93

Table S2. Sitemap predicted pocket parameters for the trajectory cluster centroids of ATP bound crystal structure (5YA1 or CS-Open-ATP).

Cluster Pocket Number	Size	Volume
Pocket 1	40	190.70
Pocket 2	35	146.80
Pocket 3	51	243.53
Pocket 4	55	294.637

Table S3. Sitemap predicted pocket parameters for the trajectory cluster centroids of ADP bound crystal structure (5YA2 or CS-Open-ADP).

Cluster Pocket Number	Size	Volume
Pocket 1	19	112.16
Pocket 2	59	284.69
Pocket 3	44	177.67
Pocket 4	31	133.08
Pocket 5	49	227.40

Table S4. Sitemap predicted pocket parameters for the trajectory cluster centroids of HM-Open-ATP.

Cluster Pocket Number	Size	Volume
Pocket 1	31	245.24
Pocket 2	66	297.72
Pocket 3	87	332.71
Pocket 4	109	380.73
Pocket 5	98	410.22

Table S5. Sitemap predicted pocket parameters for the trajectory cluster centroids of HM-Closed-ADP model.

Cluster Pocket Number	Size	Volume
Pocket 1	84	132.05
Pocket 2	158	227.75
Pocket 3	145	209.57
Pocket 4	156	261.70
